# Supplementary material for: Wavelet-based approach for diagnosing attention deficit hyperactivity disorder (ADHD)
Source: Sci Rep. 2022 Dec 19;12:21928. doi: 10.1038/s41598-022-26077-2 (PMC9763347; doi:10.1038/s41598-022-26077-2)
Supplement: Supplementary file 1 — Supplementary Information. [file 41598_2022_26077_MOESM1_ESM.pdf]

1 Impact of Task Performance on ADHD

The task performance behavioral analysis is an important aspect of this study, but because of the space constraints, we decided to discuss it in the supplementary information.

Fig. 1 and 2 show the task performance across groups with respect to average response times, standard deviations of response times, and proportion of correct responses. While the distribution of average response times is similar across groups, children with ADHD can exhibit more variability in response times and lower accuracy compared to children without ADHD. In Fig. 3, it also appears the sample of children without ADHD is older compared to the sample of ADHD children, and older children tend to perform more accurately with less variability in response times than younger children in this sample.

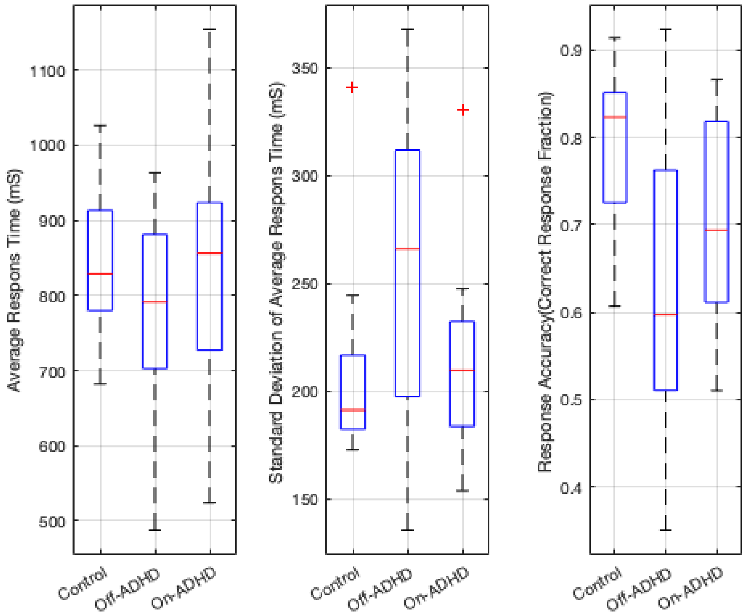

Figure 1. Variability in performance measures: Average response time, standard deviation of response time, and proportion of correct responses (number of correct answers/160), where ADHD (no med) represents ADHD children off medication and ADHD(med) represents ADHD children on medication.

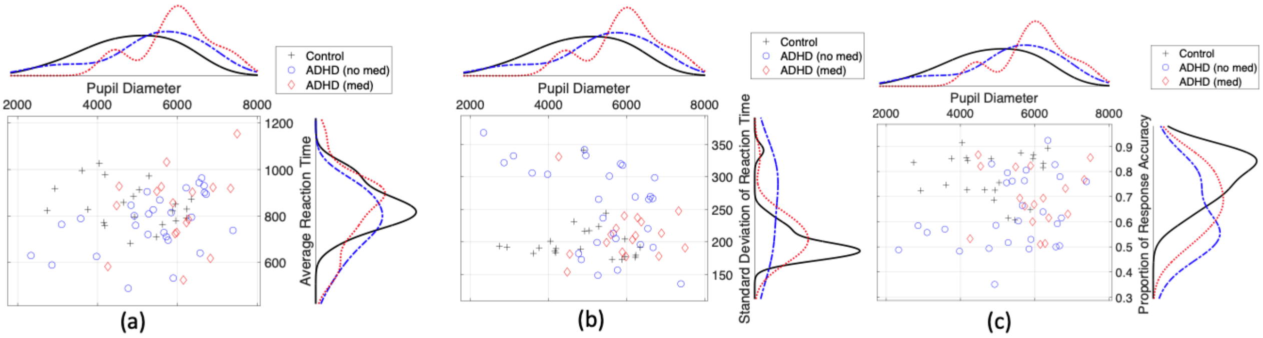

Figure 2. Scatter plots of average pupil diameter over 160 trials with the three performance measures, average response time, standard deviation of response time, and proportion of correct responses.

As part of the investigation into the contribution of these performance behaviors in detecting ADHD, the standard deviation of response times and the proportion of correct responses were included along with the classification features derived from feature engineering and self-similarity-based approaches. There was a slight improvement in classification performance for both approaches with the inclusion of these factors, as shown in Table 1. This could be due to age differences between groups, which may have affected cognitive measures, particularly the response accuracy. However, even after controlling for task performance, models using the proposed features still outperformed those using features derived from feature engineering in the original data domain.

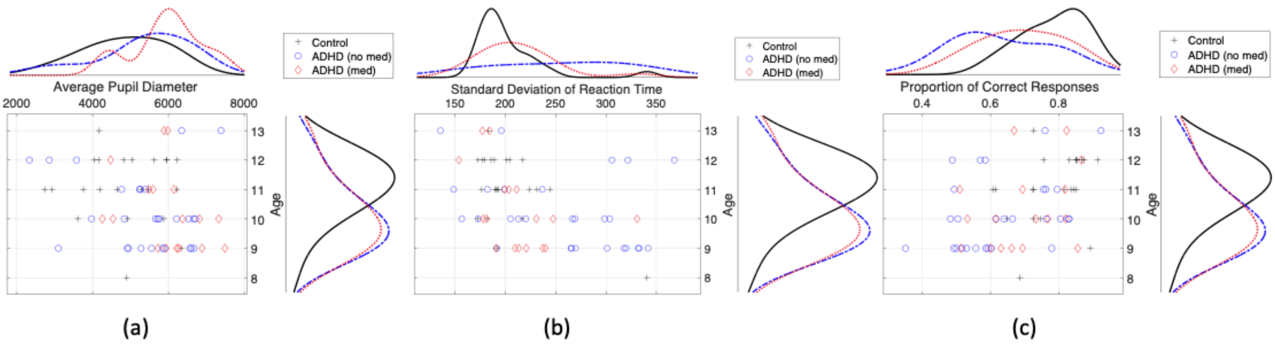

Figure 3. Scatter plots of average pupil diameter over 160 trials, standard deviation of response time, and proportion of correct responses with age. Note the difference in ages between cases and controls.

Classification Method	Classification Model					
	LR		SVM		KNN	
	Sensitivity	Specificity	Sensitivity	Specificity	Sensitivity	Specificity
**Self-similarity**	**0.89**	**0.81**	**0.90**	**0.79**	**0.93**	0.73
Feature Engineering	0.71	0.73	0.75	0.73	0.75	**0.72**
	Accuracy		Accuracy		Accuracy	
**Self-similarity**	**84.64**		**84.33**		**82.73**	
Feature Engineering	71.60		74.21		73.92	

Table 1. Classification performance with the integrated features from self-similar behavior-based method and task performance measures using data from ADHD (no med) and Control groups.

2 Impact of Medication on ADHD Detection

This is another analysis that we conducted, but did not included in the main manuscript is the impact of medication of methylphenidate on ADHD detection.

The results of controls vs ADHD (med) data analysis outcomes are summarized in Table 2. They indicate that the proposed self-similarity-based method classifies cases and controls better than the feature engineering method. When comparing performance of the proposed approach between ADHD(med) and ADHD(no med) groups (see Table 1 in the main manuscript and Table 2), the on-medication group shows a slight improvement while the feature engineering method does not result in a significant change.

In fact, the administration of methylphenidate improves ADHD symptoms and hence we expected a decline in classification performance. Surprisingly, our analysis shows the opposite behavior. This could be due to several reasons. For example, using a 24-hour time gap, children with ADHD performed visual tasks with and without medication. In order to achieve significant improvement in symptoms, this time gap may not be sufficient. Also, methylphenidate is generally effective in improving symptoms of ADHD at an early stage. In the analysis of the data, it was assumed that the stage of ADHD was the same in all cases. However, there is no information on the stage of development the ADHD-diagnosed subjects were in at the time. Although the administration of methylphenidate resulted in improving performance behaviors (higher average response time and proportion of response accuracy in Fig. 1), we do not observe a significant influence on pupil diameter spectral dynamics and average response time.

Overall, it appears that medication did not affect the discriminatory properties of wavelet-based descriptors in general. Further studies are needed to characterize the influence of medication on pupillary dynamics.

Classification Method	Classification Model					
	LR		SVM		KNN	
	Sensitivity	Specificity	Sensitivity	Specificity	Sensitivity	Specificity
**Self-similarity**	**0.82**	**0.81**	**0.82**	**0.93**	**0.75**	0.95
Feature Engineering	0.64	0.74	0.64	0.73	0.78	0.52
	Accuracy		Accuracy		Accuracy	
**Self-similarity**	**81.48**		**87.52**		**85.20**	
Feature Engineering	69.13		68.52		65.39	

Table 2. Classification performance of the self-similar behavior-based method with ADHD (med) data with controls (1024 window size, and six features and wavelet decomposition levels).
